# Subclinical hypothyroidism and insulin resistance in polycystic ovary syndrome: is there a relationship?

**Published:** 2014-07

**Authors:** Anahita Enzevaei, Saghar Salehpour, Maryam Tohidi, Nasrin Saharkhiz

**Affiliations:** 1 *Infertility and Reproductive Health Research Center, Shahid Beheshti University of Medical Sciences, Tehran, Iran. *; 2 *Prevention of Metabolic Disorders Research Center, Research Institute for Endocrine Sciences, Shahid Beheshti University of Medical Sciences, Tehran, Iran.*

**Keywords:** *Polycystic Ovary Syndrome*, *hypothyroidism*, *Insulin resistance*

## Abstract

**Background: **Polycystic ovary syndrome (PCOS) is the most common hyperandrogenic disorder among women and is often defined as hyperandrogenic syndrome. These patients are at risk for oligo/amenorrhea, chronic anovulation, infertility, obesity, spontaneous abortion, insulin resistance, hyperinsulinemia and metabolic syndrome. Thyroid disorders especially hypothyroidism is more common in these people. In PCOS patients, subclinical hypothyroidism may aggravate insulin resistance.

**Objective: **The goal was to find any relationship between subclinical hypothyroidism and insulin resistance in PCOS patients.

**Materials and Methods: **In this prospective cross sectional study we included all PCOS patients coming to infertility clinic of Taleghani Hospital in 2010-2012 who had the criteria of Rotterdam for PCOS. Then the clinical examination was done for them and height, weight, body mass index and lab data were measured including thyroid hormone and biochemical profile. The data were analyzed by SPSS software version 20.

**Results: **Among 75 PCOS patients, 19 (25.5%) had subclinical hypothyroidism and 56 patients (74.4%) were euthyroid. The prevalence of insulin resistance was 22.7% and 77.3% of patients had no insulin resistance were normal.

**Conclusion: **We could find no relationship between insulin resistance and subclinical hypothyroidism in PCOS patients.

## Introduction

Polycystic ovary syndrome (PCOS) is the most common hyperandrogenic disorder, characterized by oligo/anovulation and menstrual disturbances leading to infertility and early abortion (1, 2). The main endocrine derangements responsible for the clinical manifestations are hyperandrogenemia and abnormal insulin response to glucose. About 50-70% of these patients are insulin resistant and suffer from metabolic syndrome that predisposes them to diabetes mellitus and cardiovascular diseases (2, 3). Hypothyroidism has been shown to cause many metabolic derangements, such as decrease in glucose disposal or its uptake by muscles or adipose tissues in response to insulin, increase in the level of sex hormone-binding globulin, weight gain, and hyperlipidemia, all of which can lead to insulin resistance (1, 4, 5). 

In particular it has been extensively demonstrated that thyroid hormones, and specifically T3, have insulin antagonistic effects at the liver level that lead to an increased glucose hepatic output, via an enhanced rate of gluconeogenesis and glycogenolysis (6). For this reason all the existing criteria used for diagnosis of PCOS necessitate exclusion of hypothyroidism at first (2, 7). The relationship between insulin resistance and hypothyroidism has been studied for decades, but similar effects of subclinical hypothyroidism (SCH) on various clinical and metabolic parameters have not received much attention in these patients (4, 8, 9). SCH is defined as a serum thyroid stimulating hormone (TSH) above the defined upper limit of the reference range, with a serum free thyroxin (fT4) within the reference range.

Mueller *et al* recently demonstrated an increase in insulin resistance in a subgroup of euthyroid women with PCOS having TSH >2 IU/L in comparison with those having TSH <2 IU/L (1). These findings have not been shown in many other studies. In PCOS patients, subclinical hypothyroidism may aggravate the insulin resistance; therefore it may be appropriate to screen for thyroid problems in these patients (1). Regarding the insufficiency of literature on this subject and the inconsistency of the existing data, the present study tries to assess the relationship between SCH and PCOS clinical and biochemical phenotype in a group of Iranian PCOS patients. On the other hand we are trying to find a cut off for TSH level above which the subclinical hypothyroidism is defined and assess the relationship of HOMA-IR and subclinical hypothyroidism.

## Materials and methods

In this prospective cross-sectional study we included all PCOS patients attending to infertility clinic of Taleghani Hospital, Shahid Beheshti University of Medical Sciences (SBUMS), Tehran, Iran, between February 2010 to February 2012. The included patients signed an informed consent explaining the purpose of the study. This study was approved by ethical committee of Research council of SBUMS as a residency thesis. PCOS was diagnosed according to the criteria of the Rotterdam European Society of Human Reproduction and Embryology-American Society for Reproductive Medicine-sponsored PCOS consensus workshop group (7). Patients with two out of the following three features were considered as PCOS: oligoovulation and/or anovulation, clinical and/ or biochemical signs of hyperandrogenism, and polycystic ovaries on ultrasound examination (the presence of 12 or more follicles measuring 2-9 mm in diameter). Oligo/anovulation was defined as the presence of oligomenorrhea (menstrual cycles of >35 days) or amenorrhea (lack of menstrual period for 6 months or more).

**Exclusion Criteria**

Patients who had known history of diabetes mellitus, hyperprolactinemia, congenital adrenal hyperplasia, hypothyroidism or hyperthyroidism, Cushing’s disease, renal, hepatic, or cardiac dysfunction, a history of ovarian or adrenal neoplasm, and those using any medication (e.g. oral contraceptives, insulin-sensitizing drugs, statins, radioactive Iodine, Levothyroxine, corticosteroids and GnRH agonists and antagonists) within 6 months of the enrolment to the study were excluded. 

**Measurements**

Complete systemic clinical examination and anthropometric assessment including height, weight, body mass index (BMI), waist and hip circumferences was done for every patient by a single person and with the same instruments in the clinic so that the inter observer errors were limited, and lab data were measured including thyroid function tests, fasting insulin, HOMA-IR index of insulin resistance, and hormonal and biochemical profile. Insulin resistance, defined by the homeostasis model assessment insulin index (HOMA-IR), was calculated using the following equation:

HOMA-IR= Fasting insulin (μU/L) × Fasting glucose (mmol/L)/22.5 (2).

The value of HOMA-IR greater than 3.2 was assumed as insulin resistance. TSH level >3.75 IU/L (according to laboratory normal range) with normal free T4 and free T3 were assumed as subclinical hypothyroidism.

**Laboratory Measurements**

After 12 hours overnight fasting, a blood sample was drawn into vacutainer tubes from the antecubital region. The collected serum samples were immediately frozen at -80ºC for subsequent analyses. Fasting serum glucose, triglycerides (TG), total cholesterol (TC), high-density lipoprotein cholesterol (HDL-C) and low- density lipoprotein- cholesterol (LDL-C) were measured using the enzymatic colorimetric method (Pars Azmon Inc., Tehran, Iran) by a Selectra 2 auto-analyzer (Vital Scientific, Spankeren, The Netherlands). Very low- density lipoprotein cholesterol (VLDL- C) was calculated using the related formula. In all the biochemical analyses, the intra- assay coefficients of variation (CVs) were less than 2.0%.

Serum free thyroxin (FT4), free triidothyronine (FT3) and insulin were assayed by the electrochemiluminescence immunoassay (ECLIA) method, using Roche Diagnostic’s kits and the Roche/Hitachi Cobas e-411 analyzer (GmbH, Mannheim, Germany); the intra- assay CVs in these hormone assays were below 1.8%. Serum thyroid stimulating hormone (TSH), follicle stimulating hormone (FSH) and luteinizing hormone (LH) were measured by immune-radiometric assay (IRMA) using commercial kits (Izotop, Budapest, Hungary) and a Dream Gamma- 10 gamma counter (Shin Jin Medics Inc., Korea); the intra-assay CVs in all IRMA runs were less than 3.4%.

Enzyme-linked immunosorbent assay was used for determination of progesterone, estradiol, free testosterone, sex hormone binding globulin (SHBG) using dbc kits (Diagnostic biochem Canada Co., Ontario, Canada) and the Sunrise ELISA reader (Tecan Co., Salzburg, Austria) with CVs less than 5.0% in all ELISA runs. Lyophilized quality control material (Lyphochek Immunoassay plus Control, Bio-Rad Laboratories) was used to monitor accuracy of the all above mentioned hormone assays. The normal biochemical range for TSH according to our laboratory measures is 0.3-3.75 mIU/L.

Statistical analysis

The data are presented as mean±SD and were analyzed with SPSS for Windows 20 package, using the Student’s t-test for comparing means between two groups, Chi Square test for nominal variables, and Pearson’s Correlation test for assessment of correlation between TSH and HOMA-IR (index of insulin resistance). P<0.05 was considered statistically significant.

## Results

In this study, 75 PCOS patients were enrolled among which 19 patients (25.5%), had TSH>3.75 and normal levels of FT3 and FT4 who are known to suffer from SCH. On the other hand, 56 patients (74.4%) were euthyroid. Insulin resistance frequency was observed in 17 cases (22.7%) based on the HOMA-IR >3.2, and 58 patients (77.3%) had no insulin resistance. The average age of the patients was 26±4.2 years which showed no significant difference in the two groups of those with SCH and those with Euthyroidism. Also, anthropometric parameters such as weight, height, BMI, waist circumference, waist to hip ratio were not significantly different between group 1 (Euthyroidism) and group 2 (SCH) (Table I). 

In addition, in euthyroid group the mentioned parameters were analyzed in subgroups of TSH (TSH>2, TSH>2.5, TSH>3) and in none of them no significant difference were seen between groups. The average level of TSH was 1.89±0.78 in euthyroid group and 6.89±5.52 in SCH group which showed significant increase in SCH group (p=0.006). But FT3 and FT4 level were similar in the two groups and were both in the normal range. Comparing the two groups, fasting glucose levels and fasting insulin levels were similar and there was no significant difference in the insulin resistance index. Although serum lipids had a small clinical difference in two groups, but the difference was not significant. Other serum lipoproteins and cholesterol didn’t show significant differences between the two groups (Table II). 

Even after Pearson’s correlation test was performed, TSH levels did not correlate with cholesterol, serum lipids and triglyceride levels (p=0.09). To evaluate the relationship between hormones and SCH, no significant difference was found between the levels of estradiol, progesterone, LH, FSH, and the LH/FSH ratio in the two groups, but the testosterone level in the euthyroid group was slightly higher than that of the SCH group. This finding was statistically significant (1.05 pg/mL in SCH group compared to 1.5 pg/mL in euthyroid group; p=0.006) (Table II). 

In clinical symptoms perspective, such as acnes, hirsutism, alopecia, virilization, truncal obesity, galactorrhea, hyperpigmentation, and infertility history there was no significant difference between the two SCH and euthyroid groups. There were more irregularities in menstrual cycles among euthyroid PCOS patients (79.6% in euthyroid group vs. 42.1% in SCH group) (p=0.002). In the euthyroid group, the patients were categorized into subgroups with TSH >2, TSH >2.5 and TSH >3 in none of which the clinical, hormone, and biochemical parameters had significant differences. The patients were then studied in terms of insulin resistance based on HOMA-IR index. The prevalence of insulin resistance was 22.7%.

According to the aforementioned index, the patients were divided into two normal and insulin resistant groups. In the former, mean BMI was 25±4.31 while in the latter, it was 27.7±3.7 which are clinically different, and however, it was not statistically significant. The waist/hip ratio did not show significant difference between the two groups but the average waist circumference was 90.9±10.04 in insulin resistant group and 83.1±10.2 in the normal group which showed a significant difference (p=0.008). Among the lipid profile parameters, TG and VLDL levels in the two normal and insulin resistant groups had significant differences. In the insulin resistant group, triglyceride level was 159.1±74.3 and in the normal group it was 94.7±40.8 (p=0.000) and VLDL level was 31.7±14.8 in the insulin resistance group, while it was 18.9±8.1 in the normal group (p=0.000). But the total cholesterol level and HDL did not show significant differences in the two groups. Regarding clinical symptoms, such as acnes, hirsutism, alopecia, virilization, truncal obesity, galactorrhea, hyperpigmentation, and infertility history, there was no significant difference between the two groups of insulin resistance and normal.

**Table I:** Comparison of anthropometric parameters in the two groups of subclinical hypothyroidism and euthyroid (student’s T- test)

	**Euthyroid**	**Subclinical hypothyroidism (SCH)**	**p-value**
Age	25.62 ± 3.93	27.21 ± 5.12	0.458
Weight	66.66 ± 11.4	63.47 ± 10.3	0.641
Height	161 ± 0.06	159 ± 0.07	0.456
Body Mass Index (BMI)	25.7 ± 4.39	24.9 ± 3.97	0.523
Waist circumference	84.83 ± 10.8	84.78 ± 10.4	0.965
Waist/hip ratio	0.82 ± 0.08	0.83 ± 0.07	0.471

We found no significant relationships in these anthropometric parameters between normal and SCH groups

**Table II:** Comparison of hormone profile and insulin resistance indices among the two groups of euthyroid and subclinical hypothyroidism (Student’s T- test)

	**Euthyroid**	**Subclinical hypothyroidism**	**p-value**
TSH	1.89 ± 0.78	6.89 ± 5.52	0.006
Insulin	11.45 ± 6.87	10.65 ± 5.30	0.709
FBS	90.03 ± 10.04	87.21 ± 7.06	0.384
HOMA-IR	2.52 ± 1.44	2.29 ± 1.12	0.739
Cholesterol	182.42 ± 30.30	170.84 ± 30.4	0.643
TG	115.58 ± 60.14	91.05 ± 41.26	0.116
HDL	55.05 ± 13.05	59.15 ± 13.96	0.307
LDL	99.01 ± 21.17	90.26 ± 19.37	0.446
VLDL	23.07 ± 11.96	18.31 ± 8.32	0.123
LH	10.11 ± 8.46	9.21 ± 7.53	0.805
FSH	8.22 ± 2.24	8.03 ± 2.70	0.217
Progesterone	2.19 ± 3.89	1.66 ± 2.75	0.197
Estradiol	49.80 ± 27.26	55.52 ± 50.71	0.118
LH/FSH ratio	1.21 ± 0.75	1.05 ± 0.64	0.746
Free testosterone	1.5 ± 1.14	1.05 ± 0.62	0.006

## Discussion

PCOS is the most prevalent endocrine disorder among women and is generally defined in the form of hyperandrogenic syndrome. About 50-70% of these patients are hyperinsulinemic insulin resistant and suffer from metabolic syndrome which by itself increases the risk of type II diabetes and cardiovascular disorders. The correlation between resistance to insulin and hypothyroidism has been given significant attention for decades and extensive studies have been carried out in this regard. In this study, 75 patients suffering from PCOS were studied out of which 25.5% were diagnosed to have SCH. 

In comparison with other studies in the literature such as the one undertaken by Michalakis e*t al* the prevalence of SCH was 23% among patients consulting for infertility, and 17.5% among those with PCOS in another study (10). In a similar work by Maratou *et al* insulin resistance was also reported among some patients with SCH, and it was observed in both fasting and non-fasting cases, however it did not transform into overt fasting hyperglycemia (11). Roos *et al* observed interesting increase in the level of insulin resistance parameters even under minute drops in thyroid hormone levels (12). These findings bring to mind the question that does the hypothyroidism intensity have additional impact over insulin resistance? And is this insulin resistance in fact due to changes in thyroid hormones?

In the research by Muller *et al* insulin resistance in patients with TSH ≥2 was more common than those with TSH <2 even after the results were adjusted for BMI. Also in a similar work by the same group, it was observed that if TSH >2.5, fasting insulin resistance was higher than that of TSH <2.5 (1). But, in Ganie’s *et al* research conducted in India, no differences in either clinical or hormone aspects of insulin resistance were found between the two groups of euthyroidism and SCH, although there were some occasional differences which were not statistically significant (13). Similarly, in the study done by Celik *et al* in Turkey, in patients suffering from PCOS, there was no significant difference in insulin resistance parameters and fasting insulin levels between two groups of SCH and euthyroidism; however, the PCOS+SCH group had higher levels of fasting insulin as well as greater insulin resistance compared to normal people and those with euthyroidism (14).

Patients in our study were all PCOS cases who were all similar in terms of age, weight, height and BMI. Insulin resistance prevalence was 22.7% in the cases being studied. Comparatively in the study conducted by Hosseinpanah* et al* the insulin resistance was reported to be 27.2% (15). Also in the findings in the present study, no significant difference was found in the criteria (indices) of determining the Insulin resistance including HOMA-IR index, fasting insulin level, and the fasting insulin to glucose ratio in the two groups of euthyroidism and SCH. However, in findings by Celik *et al* the two groups were not comparable in BMI and waist/hip ratio which was a restriction, but after they eliminated the mentioned variables and performed complementary tests, no significant difference in insulin resistance parameters were observed. This may indicate the confounding impact of BMI and waist/hip ratio (14). The patients in the two groups in our study were quite similar; however, insulin resistance had no significant difference between the hypothyroid and euthyroid groups.

Like findings by Ganie *et al* and unlike those by Muller *et al *in this study no significant difference was observed in hormone parameters or biochemical ones associated with insulin resistance in none of the different cutoff levels -2, 2.5, 3, 3.75, 4.5-of TSH. This probably means that the TSH level, when FT3 and FT4 are at normal levels, has no significant hormonal or metabolic impact or side effect (1, 13). 

On the other hand, different studies have shown the relationship between triglyceride and LDL levels with SCH, but the results are very controversial. For instance, in the study done by Ganie *et al* the triglyceride in the SCH group had significantly higher levels than the control group, but in the study undertaken by Tuzca *et al* increase in LDL without any changes in TG and HDL in the SCH were observed in comparison with the control group (13, 15, 16). A study by Al Sayed *et al *in Kuwait had similar findings and in Brenta *et al* study no difference in lipid levels in PCOS+SCH patients has been observed comparing to the control group (17, 18). Overall, this study does not show differences in insulin resistance parameters, lipid levels and clinical appearances in patients with PCOS and SCH compared to euthyroid cases. 

## Conclusion

Due to the variety and difference in findings in various studies, it is not clear whether or not SCH has significant effects on the insulin resistance parameters. This lack of difference in insulin resistance parameters and lipid profiles between patients with SCH and those with euthyroidism may be owing to the ethnic diversities as in the studies undertaken in India, Kuwait and South-East Asia no significant difference has been observed while in the studies in Europe there have been some differences. In this regard, more extensive multi-centered researches with larger sample size are recommended. Also, a meta-analysis of the results from various studies in hand can lead to more integrated comprehensive results in the field.

## Footenote

***This article extracted from residential thesis. (Anahita Enzevaei)***
